# Editorial of Topic Issue “Chromatography–Mass Spectrometry Analysis in Biomedical Research and Clinical Laboratory”

**DOI:** 10.3390/molecules31030481

**Published:** 2026-01-30

**Authors:** Andreas Tsakalof, Constantinos K. Zacharis

**Affiliations:** 1 School of Medicine, University of Thessaly, GR-41500 Larissa, Greece; 2 Department of Pharmacy, Aristotle University of Thessaloniki, GR-54124 Thessaloniki, Greece

Throughout the 21st century, chromatography–mass spectrometry (CMS) evolved from a specialized, expert-level analytical technique to a central instrumental platform in biomedical research, and its adoption by clinical laboratories has brought about a revolution in disease diagnosis. Nowadays, CMS plays a pivotal role in cutting-edge omics research, disease biomarkers discovery and validation, metabolic pathway elucidation, personalized medicine, drug development, and clinical disease diagnosis. These research areas are unified by their use of the CMS instrumental platform, and the aim of this issue was to highlight the multidisciplinary nature of the field and to provide a forum in which researchers can present their latest findings while becoming acquainted with CMS applications across diverse scientific domains. Six MDPI journals participated, giving the authors the opportunity to select the journal that best matches the subject of their research (Figure 1). Fifty-five (55) manuscripts were submitted, and seventeen (17) were ultimately accepted for publication.

The largest number of manuscripts (six) was published in *Molecules*, which focuses on chemistry and related scientific disciplines. The published articles encompassed a wide range of biomedical applications. The study of A. Blazewicz et al. [1] combines targeted lipidomics via flow injection electrospray–tandem mass spectrometry (FI-ESI–MS/MS) with inductively coupled plasma–mass spectrometry (ICP-MS) trace element analy-sis to uncover metabolic signatures of hypothyroidism and lipidome relationship with non-occupational exposure to toxic elements. Using the multiple reaction monitoring (MRM) mode, the authors achieved sensitive and selective quantification of 145 plasma lipid species, enabling the precise characterization of alterations in lysophosphatidylcholines and phosphatidylcholines. The integration of lipidome analysis with measurements of toxic elements revealed how subtle environmental exposures and disease subtypes shape lipid metabolism in autoimmune and non-autoimmune hypothyroidism. The research of L. Molteni et al. presents a robust ultra-high-pressure liquid chromatography–tandem mass spectrometry (UHPLC–MS/MS) method for the simultaneous quantification of L-DOPA, levodopa methyl ester, and carbidopa in human plasma, providing a powerful analytical tool for therapeutic drug monitoring in Parkinson’s disease [2]. By coupling UHPLC with triple quadrupole mass spectrometer (QqQ) mass spectrometry, the method ensures high selectivity, sensitivity, and reproducibility, enabling the accurate assessment of L-DOPA pharmacokinetics under different therapeutic regimens. Its successful application to a clinical case highlights the potential of LC–MS/MS-based approaches to support personalized treatment strategies and optimize the efficacy and safety of L-DOPA therapy. A year later, a fully validated UHPLC–MS/MS method for the simultaneous quantification of cenobamate and concomitant anti-seizure medications in human plasma has been developed and validated in the same laboratory [3]. This method offers a powerful analytical tool for therapeutic drug monitoring. It ensures high sensitivity, selectivity, and robustness across a clinically relevant concentration range, in compliance with ICH M10 guidelines. This approach supports precision medicine in epilepsy by enabling accurate monitoring of drug levels during combination therapy, thereby improving the clinical management of patients with refractory and drug-resistant epilepsies. This Editorial highlights the development of a highly sensitive LC–MS/MS-based assay for the quantification of cytotoxic T-lymphocyte-associated protein 4 (CTLA-4) in human T cells, addressing a major analytical gap in immuno-oncology research [4]. By leveraging the specificity and sensitivity of liquid chromatography–mass spectrometry, the method enables the absolute quantification of CTLA-4 down to a few copies per cell, providing a powerful tool to support pharmacodynamic, efficacy, and safety assessments of CTLA-4–targeted cancer therapies across preclinical and clinical studies. The study of Y. Taya et al. [5] underscores the value of LC–MS/MS-based analysis of 4*β*-hydroxycholesterol as a sensitive biomarker for detecting even weak cytochrome P450 family 3 subfamily A member 4 (CYP3A4) induction in clinical drug development. By optimizing plasma collection and storage conditions and introducing the monitoring of a cholesterol oxidation product as a quality marker, the authors ensure accurate and reliable quantification of 4β-HC using liquid chromatography–mass spectrometry. This refined LC–MS approach enables robust evaluation of CYP3A4 induction by new chemical entities without the need for probe substrates, strengthening the role of lipid biomarkers in clinical DDI studies. Notably, all studies published in *Molecules* utilized QqQ from different vendors, reaffirming the status of QqQ as the instrument of choice in biomedical research, a point further emphasized by Tsakalof et al. in their review of QqQ applications in biomedicine [6]. By analyzing publication trends, the review shows a marked increase in QqQ use over the past decade, particularly in endocrine research, therapeutic drug monitoring, and forensic toxicology, where it is progressively replacing traditional immunoassays. The paper also discusses recent technological advances in QqQ platforms that have strengthened their clinical applicability, while positioning high-resolution accurate mass (HRAM) instruments as complementary tools driving innovation in untargeted biomarker discovery.

Four manuscripts were published in *Biomolecules*, a journal dedicated to multidisciplinary research in “chemistry, biology, molecular medicine and material sciences.” The clinical impact of LC–HRMS as a superior alternative to immunoassays for urinary free cortisol analysis has been highlighted by G. Casals et al. [7]. By exploiting the high specificity of liquid chromatography coupled with high-resolution accurate mass detection, the validated method enables the simultaneous and reliable quantification of cortisol, cortisone, 6β-hydroxycortisol, and 18-hydroxycortisol, while also allowing for untargeted compound identification. This LC–HRMS approach overcomes the major limitations of immunoassays caused by metabolite cross-reactivity, providing a more accurate and comprehensive tool for the diagnosis and follow-up of hypercortisolism and disorders of adrenal steroid metabolism. The research group of Y. Mechref showcases the power of advanced LC–MS/MS glycoproteomics to unravel variant-specific *O*-glycosylation patterns of the SARS-CoV-2 spike S1 protein [8]. By combining a double-digestion strategy with a highly sensitive LC-MS/MS workflow, the authors achieved in-depth and site-specific characterization of *O*-glycans across eleven viral variants. The approach reveals both conserved and variant-dependent glycosylation features, highlighting the exceptional O-glycosylation density and sialylation of the Omicron variant, and provides critical molecular insight into how glycan remodelling may influence immune evasion, infectivity, and vaccine design. The unique ability of advanced nano-LC-PRM/HRMS to decipher the secretion mechanisms and redox states of immunomodulatory high mobility group box 1 (HMGB)1 in response to anticancer drugs has been proven [9]. By combining immunoprecipitation, stable isotope dilution, and differential alkylation with high-resolution mass spectrometry, the authors achieved sensitive and selective quantification of HMGB1 and its post-translational modifications. The LC-MS-based approach revealed that cisplatin, unlike carboplatin, induces HMGB1 secretion through an XPO1-dependent pathway and predominantly releases the fully reduced, immunologically active form of the protein. These findings demonstrate how high-resolution targeted mass spectrometry can uncover drug-specific molecular mechanisms with direct implications for tumour biology, immune modulation, and the optimization of combined chemo-immunotherapy strategies. The combination of HPLC and LC-HRMS provides a powerful platform to assess the stability and structural integrity of mitochondrial-derived peptides such as Humanin-G [10]. By using LC-HRMS, the authors achieved precise identification and characterization of HNG degradation products, including site-specific oxidation and dimerization species, which cannot be resolved by conventional analytical techniques alone. The work highlights the essential role of high-resolution LC–MS in monitoring peptide stability, elucidating degradation pathways, and guiding optimal formulation and storage conditions for biologically active therapeutic peptides.

The *Journal of Clinical Medicine*, dedicated to clinical and preclinical research, accepted three submitted studies related to the scope and aims of the journal. Meinarovich et al. propose a multimodal risk assessment algorithm for post-cardiac surgery complications, combining clinical data with metabolomic profiles and sepsis-related biomarkers and using gas chromatography coupled with a single quadrupole mass analyser (ISQ, Thermo Electron Corporation, Santa Clara, CA, USA) for microbiome metabolite quantification in patients’ blood [11]. The second study by Kośliński et al. reveal the significant differences in amino acids metabolic profiles in serum between patients with multiple sclerosis and myasthenia gravis and healthy controls, thus forming the groundwork for the development of a clinical assay for the differential diagnosis of these autoimmune neurological diseases [12]. The target amino acids were quantified by liquid chromatography coupled with a triple quadrupole mass spectrometer LCMS-8045 (Shimadzu, Kyoto, Japan). Amino acids homeostasis was also investigated by Posma et al. in patient with metformin intoxication to clarify the biochemical basis of intoxication [13]. In this study, the amino acids and other metabolites were quantified in plasma, urine, and dialysate samples by gas chromatography coupled to a single quadrupole ISQ mass analyser (ThermoFisher, Dreieich, Germany).

Two studies were published in the *International Journal of Molecular Sciences*, which provides a forum for research in molecular medicine, molecular and cellular biology, and biochemistry. The biochemical pathways involved in hepatic encephalopathy (HE), a central nervous dysfunction syndrome, were investigated on animal models (mice) by Guo et al. using combined metabolomic and proteomic profiling in serum samples [14]. High-resolution hybrid Orbitrap mass analysers (Q Exactive™ HF and Q Exactive™ HF-X, Thermo Fisher Scientific, Waltham, MA, United States) with UHPLC liquid chromatography systems were used in this study for the quantification of 50 metabolites and 226 proteins. The acquired analytical data enabled the identification of altered metabolic pathways in HE. The study underscores the capacity of multi-omics approaches to reveal the complex biochemical mechanisms that drive disease pathophysiology, as well as their potential in disease biomarkers discovery. The second study, by Ahn et al., focuses on optimizing sample pre-treatment for the subsequent headspace GC-MS quantification of volatile organic compounds (VOCs) in whole canine blood [15]. With the overarching aim of minimizing matrix effects and enhancing analytical sensitivity, the authors developed an optimized protocol that improves VOC recovery and measurement reliability. The analytical setup employed in this study consisted of a Trace 1610 gas chromatograph coupled with an ISQ 7610 single-quadrupole mass spectrometer and a TriPlus 500 headspace autosampler (Thermo Fisher Scientific, Waltham, MA, USA).

The journal *Separations* accepted two publications dedicated to development of new approaches for the analysis of biologically important compounds and metabolites in biological fluids. In the study of Hefni et al., the UPLC-MS/MS method was developed for the simultaneous quantification of nutritional quaternary ammonium compounds (betaine, L-carnitine, acetyl-L-carnitine, and propionyl-L-carnitine) and their downstream metabolites (trimethylamine, trimethylamine-N-oxide) in plasma [16]. The terminal metabolite of this pathway, trimethylamine-N-oxide, is associated with various chronic diseases that mainly are consequences of metabolic syndrome. The developed assay is intended to have clinical value in the monitoring of this pathway in patients with metabolic syndrome. The study by Kim et al. presents a new LC-MS/MS method for the indirect, after-dephosphorylation quantification of signalling lipid sphingosine-1-phosphate (S1P) in plasma and kidney tissues obtained from a chronic kidney disease (CKD) mouse model [17]. The liquid chromatography-AB Sciex QTRAP 3200 mass spectrometer (SCIEX, Toronto, Canada) was used for analyte quantification.

In conclusion, we would like to underline that the papers presented above originate from different scientific domains, but they are unified by their common use of the chromatography–mass spectrometry (CMS) platform. Grouping them within a single thematic topic highlights the multifaceted potential of CMS and demonstrates its broad applicability across both biomedical research and clinical practice. Additionally, presenting them as a unified topic allows the readership of each contributing journal to gain exposure to related studies published in the other journals represented in this collection, thereby enhancing cross-disciplinary visibility.

We wish to sincerely thank all the authors for their outstanding contributions to this topical issue. Their high-quality studies demonstrate the depth and continuous innovation of CMS applications in biomedical and clinical research, ranging from therapeutic drug monitoring and biomarker discovery to proteomics, lipidomics, steroid analysis, and glycoproteomics. Through the development and validation of robust LC/GC–MS- and HRMS-based methodologies, the authors have highlighted the essential role of advanced analytical platforms in improving diagnostic accuracy, understanding disease mechanisms, and supporting precision medicine. Their collective efforts not only advance the state of the art in analytical and clinical mass spectrometry but also provide valuable reference frameworks for future research and clinical implementation.

## Figures and Tables

**Figure 1:**
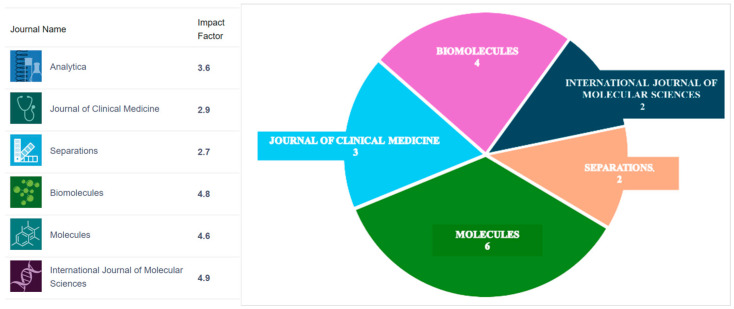
Participating journals and the distribution of the published manuscripts.

## Data Availability

No new data were created or analyzed in this study. Data sharing is not applicable to this article.
